# Enhanced Delivery of Imatinib into Vaginal Mucosa via a New Positively Charged Nanocrystal-Loaded in Situ Hydrogel Formulation for Treatment of Cervical Cancer

**DOI:** 10.3390/pharmaceutics11010015

**Published:** 2019-01-04

**Authors:** Li-qian Ci, Zhi-gang Huang, Feng-mei Lv, Jun Wang, Ling-lin Feng, Feng Sun, Shui-juan Cao, Zhe-peng Liu, Yu Liu, Gang Wei, Wei-yue Lu

**Affiliations:** 1 Department of Pharmaceutics, School of Pharmacy, Fudan University & Key Laboratory of Smart Drug Delivery (Fudan University), Ministry of Education, Shanghai 201203, China; ciliqian@foxmail.com (L.-q.C.); 16211030018@fudan.edu.cn (Z.-g.H.); weigang@shmu.edu.cn (G.W.); wylu@shmu.edu.cn (W.-y.L.); 2 School of Medical Instrument and Food Engineering, University of Shanghai for Science and Technology, Shanghai 200093, China; 15216806348@163.com (F.-m.L.); doudou19940424@163.com (J.W.); zhepengliu@126.com (Z.-p.L.); 3 NHC Key Laboratory of Reproduction Regulation (Shanghai Institute of Planned Parenthood Research), Fudan University, Room 904, No 1 Research Building, 2140 Xietu Road, Shanghai 200032, China; fenglinglinxin@163.com; 4 Chinese Academy of Sciences Shanghai Institute of Materia Medica, Shanghai 201203, China; sunfengbbb@simm.ac.cn; 5 Experimental Teaching Center, School of Pharmacy, Fudan University, Shanghai 201203, China; chaoshui2011@126.com

**Keywords:** mucoadhesiveness, cervicovaginal tumors, cationic functionalization, imatinib, nanocrystals, in situ hydrogel

## Abstract

The present study was carried out to investigate the potential of cationic functionalization on imatinib nanocrystals to improve the mucoadhesiveness and, thus, delivery to the lesion of cervicovaginal tumors. Amino-group-functionalized imatinib nanocrystals (NC@PDA-NH_2_) were prepared with near-spheroid shape, nanoscale size distribution, positive zeta potential, and relatively high drug content with the aid of the polydopamine-coating technique. Efficient interaction between NC@PDA-NH_2_ and mucin was proven by mucin adsorption which was related to the positive zeta-potential value of NC@PDA-NH_2_ and the change in the size distribution on mixing of NC@PDA-NH_2_ and mucin. Cellular uptake, growth inhibition, and apoptosis induction in cervicovaginal cancer-related cells demonstrated the superiority of NC@PDA-NH_2_ over unmodified nanocrystals. For practical intravaginal administration, NC@PDA-NH_2_ was dispersed in Pluronic F127-based thermosensitive in situ hydrogel, which showed suitable gelation temperature and sustained-release profiles. In comparison with unmodified nanocrystals, NC@PDA-NH_2_ exhibited extended residence on ex vivo murine vaginal mucosa, prolonged in vivo intravaginal residence, and enhanced inhibition on the growth of murine orthotopic cervicovaginal model tumors indicated by smaller tumor size, longer median survival time, and more intratumor apoptosis with negligible mucosal toxicity. In conclusion, cationic functionalization endowed NC@PDA-NH_2_ significant mucoadhesiveness and, thus, good potential against cervicovaginal cancer via intravaginal administration.

## 1. Introduction

Cervical cancer is the fourth most common cause of death from cancer in women [1]. With the development and popularization of regular Papanicolaou (Pap) tests, the majority of cervical cancer patients can be diagnosed in the early stages when the tumor is small in size and confined to the cervix. Nevertheless, the current treatment for early-stage cervical cancer usually involves significant adverse side effects. Surgery and radiation therapy are associated with increased risks of future negative gynecological and obstetric outcomes, including impaired sexual function, infertility, preterm delivery, and low birth weight. Systemic pharmacotherapy is generally the last option because only a negligible proportion of the drug reaches the vaginal mucosal epithelium. Now, clinicians are realizing that cervicovaginal tumors may represent the most important potential field needing good vaginal formulations [2]. Sustained and localized drug delivery in the female reproductive tract may avoid adverse effects associated with systemic administration, improve efficacy by assuring adequate local drug concentration, and allow convenient self-administration [3]. For example, the first tyrosine kinase inhibitor, imatinib, was reported to prevent the activation of platelet-derived growth factor receptor (PDGFR), which is often overexpressed in cervical cancer [4], thus inhibiting cervical cancer cell proliferation. In a study which screened carbo- and cisplatin, topotecan, paclitaxel, imatinib, gefitinib, cetuximab, and trastuzumab on freshly isolated tumor cells of 16 patients, 66% of tumor samples were sensitive to imatinib [5]. When administered orally, it worked effectively in the clinic, possibly due to its high plasma protein binding and insufficient drug delivery to the vaginal mucosal epithelium. It is reasonable to expect a positive outcome for imatinib if vaginal formulations with adequate vaginal mucosal delivery efficiency are available.

Recently, nanocrystals (NC) drew increasing interest in field of delivery of poorly soluble drugs [6,7,8] for their nanoscale and high drug content. These properties seem to be attractive for vaginal formulations. Only nanocrystals with suitable mucoadhesive modification can overcome the self-cleaning mechanism of the vagina, assure prolonged contact time with mucosa, and avoid multiple daily doses [9]. One of the most promising mucoadhesive strategies is cationic modification, which is based on the electrostatic interaction with negatively charged mucin in the mucus, as well as mucosal epithelial cells. The most exciting recent advance is positively charged 10-hydroxycamptothecin-loaded nanogels for intravesical instillation which successfully prolonged intravesical residence time, improved penetration into the vesical mucosa, and enhanced the anti-tumor efficacy in a murine orthotopic bladder cancer model [9]. Cationic functionalization was also applied in vaginal formulations. We previously synthesized a cationic derivative of Pluronic F127 to prepare a new type of thermosensitive/mucoadhesive in situ hydrogel, which significantly prolonged intravaginal drug residence and improved mucosal penetration [10]. Similar benefits were also reported for imiquimoid-loaded nanocapsules [11].

Being highly dispersed in nature, NCs are thermodynamically unstable, necessitating surface modification to prevent possible particle growth. In the meantime, surface modification can change the in vivo performance of nanocrystals. Polyethylene glycol (PEG)ylated paclitaxel nanocrystals showed good stability and in vivo tumor inhibition efficacy [12]. Albumin-stabilized paclitaxel nanocrystals showed enhanced uptake by SPARC^+^ B16F10 melanoma cells and in vivo anti-tumor efficacy [13], and albumin-stabilized hydroxycamptothecin nanocrystals showed enhanced intratumor accumulation and prolonged survival time in MCF-7 model tumor-bearing mice [14]. Pluronic F68-stabilized paclitaxel nanocrystals exhibited elevated interaction with cancer cells and intratumor accumulation [15,16]. Pluronic F68 and soybean lecithin-stabilized 7-ethyl-10-hydroxycamptothecin nanocrystals inhibited the growth of MCF-7 model tumors more efficiently [17]. The abovementioned surface modification examples for nanocrystals are mainly based on either physical adsorption of stabilizers or chemical derivatization of the prototype drug.

Alternatively, polydopamine (PDA) coating may provide another choice. In a weak basic environment, dopamine can self-polymerize on the surface of solid materials to form a PDA layer which can subsequently react with thiol or amine groups at *o*-quinone moieties [18]. Since the pioneer work of Messersmith’s group [19], this property of PDA attracted increasing interest for its good biocompatibility and capability of forming of nanostructures [20,21] or their surface functionalization [22,23]. Liang et al. recently reported the promising performance of tumor-targeting modified PDA-coated camptothecin nanocrystals [24].

Herein, positively charged polydopamine-coated imatinib nanocrystals (NC@PDA-NH_2_) were prepared and characterized with respect to basic physicochemical properties, drug content and release, and in vitro interaction with mucin and cervical cancer-related cell lines. NC@PDA-NH_2_ was further dispersed in a Pluronic F127-based in situ hydrogel vehicle to allow adequate interaction time between nanocrystals and mucosa. The NC@PDA-NH_2_-loaded Pluronic F127-based in situ hydrogel (NC@PDA-NH_2_/FG) was optimized based on rheological and release profiles and characterized with respect to ex vivo residence on mouse vaginal mucosa, in vivo intravaginal retention, and anti-tumor efficacy in a murine orthotopic cervicovaginal tumor model.

## 2. Methods

### 2.1. Materials and Animals

Imatinib (IMN), 1,1′-dioctadecyl-3,3,3′,3′-tetramethylindotricarbocyanine iodide (DiR), 2-(4-amidinophenyl)-6-indolecarbamindine dihydrochloride (DAPI), and 6-coumarin (C6) were purchased from Meilun Biotechnology Ltd. Co. (Dalian, Liaoning, China). Pluronic F127 (F127) was purchased from BASF Ltd. Co. (Ludwigshaften, Germany). Optical coherence tomography (OCT) was purchased from Sakura Finetek Inc. (Torrrance, CA, USA). Porcine gastric mucin (PGM) was purchased from Sigma-Aldrich Ltd. Co. (St Louis, MO, USA) and purified as described in previous literature [23]. All other chemicals were of analytical grade, purchased from Sinopharm Reagent Ltd. Co. (Shanghai, China) and used as received. Two cervical cancer-related cell lines, TC-1 and SiHa, were purchased from Fu-Heng Cell Center (Shanghai, China).

Female C57BL/6 mice were provided by Shanghai Laboratory Animal Center (Shanghai, China). All animals were allowed free access to standard food and tap water and acclimated for at least one week before use. All animal experiments were carried out in accordance with the guidelines evaluated and approved by the Ethics Committee of the School of Pharmacy, Fudan University (2015-O3-YJ-LY-0D approved on 4 March 2015).

### 2.2. Preparation of NC, NC@PDA, and NC@PDA-NH_2_

NC@PDA-NH_2_ was prepared by three steps. Firstly, a mixture of imatinib (3.5 mg) and stabilizers (3.8 mg TPGS and 7.5 mg citrate acid) was fully dissolved in 3 mL of ethanol in a round-bottom flask. Ethanol was evaporated with a rotary evaporator at 40 °C to form a thin film on the wall of the flask. Then, 5 mL of 35 mM NaHCO_3_ aqueous solution was added to the film at room temperature. After hydration by gentle mixing, NCs were formed. Secondly, 5 mg of dopamine was added to 5 mL of the abovementioned NC suspension and incubated at room temperature for 10 min on a rotating rocker. Then, the suspension was centrifuged at 4448× *g* for 15 min at room temperature to remove unreacted dopamine and obtain NC@PDA. Thirdly, the pellet was re-suspended in 5 mL of pure water containing appropriate amounts of Boc-NHCH_2_CH_2_-NH_2_ to react for at least 3 h. Then, the suspension was centrifuged at 4448× *g* for 15 min at room temperature to remove unreacted Boc-NHCH_2_CH_2_-NH_2_ and was deprotected in 3 M HCl for 3 h to obtain NC@PDA-NH_2_. DiR- or C6-labeled nanocrystals were similarly prepared except that DiR or C5 (5 µg/mL) was included in the initial mixture in the first step.

### 2.3. Characterization of NC, NC@PDA, and NC@PDA-NH_2_

For determination of drug loading (DL) and encapsulation efficiency (EE), lyophilized nanocrystals with a premeasured mass were dissolved in acetonitrile (ACN), filtered with a 0.45-μm syringe filter, diluted if necessary, and submitted to HPLC analysis on an Agilent 1100 HPLC system (Palo Alto, CA, USA) equipped with an Xtimate ^®^ C18 column (25 cm × 4.6 mm, particle size: 5 μm; Welch Technology, Shanghai, China) with a mobile phase 45:55 (*v*/*v*) mixture of ACN/ammonium acetate buffer solution (10 mM, pH 10) at a flow rate 0.7 mL/min and a detection wavelength of 272 nm.

EE and DL were respectively calculated according to the following equations:

$$\mathrm{EE}\left(\%\right)=\frac{\mathrm{Amount}\;\mathrm{of}\;\mathrm{drug}\;\mathrm{in}\;\mathrm{nanocrystals}}{\mathrm{Total}\;\mathrm{amount}\;\mathrm{of}\;\mathrm{feeding}\;\mathrm{drug}}\times 100\%,$$

$$\mathrm{DL}\left(\%\right)=\frac{\mathrm{Amount}\;\mathrm{of}\;\mathrm{drug}\;\mathrm{in}\;\mathrm{nanocrystals}}{\mathrm{Total}\;\mathrm{mass}\;\mathrm{of}\;\mathrm{nanocrystals}}\times 100\%.$$

In vitro release of imatinib from nanocrystals was investigated using the dialysis bag method with vaginal fluid simulant (VFS) as the release medium, prepared as reported previously [25] The morphology of various nanocrystals was observed using a transmission electron microscope (TEM, JEM-2100F, JEOL Co., Tokyo, Japan). The size distribution and zeta potential of nanocrystals were measured at 37 °C using dynamic light scattering (DLS) with a Zetasizer (ZS-10-82, Malvern Instruments Ltd. Co., Malvern, UK).

X-ray powder diffraction (XRPD) of nanocrystals was analyzed with a D2 Phaser diffractometer (BrukerCorp., Billerica, MA, USA) with a Cu-Kα radiation source and a LYNXEYE^TM^-compound silicon strip detector. The powder patterns were obtained from 1 to 40° 2θ at a scan speed of 5°/min and a step size of 0.02°. The voltage and current used were 40 kV and 44 mA, respectively.

Differential scanning calorimetry (DSC) was carried out using a Perkin-Elmer Pyris 1 DSC instrument (Waltham, MA, USA) equipped with an intra-cooler 2P cooling accessory. Accurately weighed samples were separately sealed in standard aluminum pans and scanned from 20 to 300 °C at a heating rate of 10 °C/min with a nitrogen purge of 10 mL/min.

### 2.4. Interaction between Mucin and Nanocrystals

To investigate the change in the size distribution after mixing mucin and nanocrystals, purified PGM was mixed with NC@PDA-NH_2_ or NC at the weight ratio of 2:1 in citrate buffer (0.1 M, pH 5, the physiological vaginal pH) for DLS measurements.

For determination of the adsorption amount (AA) of mucin by nanocrystals, purified PGM was mixed with NC@PDA-NH_2_ with different positive surface charges (see Supplementary Materials) or NC at the weight ratio of 2:1 in citrate buffer (0.1 M, pH 5), incubated at 37 °C for 1 h and then centrifuged at 6672× *g* for 10 min (H1650-W centrifuge, Xiang-yi Instrument Inc., Changsha, China). The concentration of PGM in the supernatant was measured using Pierce^®^ BCA Protein Assay Kits (Thermo Scientific, Rockford, IL, USA) to calculate AA according to the following equation:

$$\mathrm{AA}=\frac{\mathrm{initial}\;\mathrm{amount}\;\mathrm{of}\;\mathrm{PGM}\;-\mathrm{amount}\;\mathrm{of}\;\mathrm{PGM}\;\mathrm{after}\;\mathrm{adsorption}}{\mathrm{Amount}\;\mathrm{of}\;\mathrm{nanocrystals}}.$$

### 2.5. Interaction of Nanocrystals with Cervicovaginal-Related Cell Lines

TC-1 and SiHa cells were grown in RPMI-1640 medium and DMEM medium, respectively, both containing 10% fetal bovine serum (FBS) and penicillin (100 IU/mL) and streptomycin (100 μg/mL).

For intracellular localization of nanocrystals, TC-1 cells were seeded in a 35-mm dish with a glass window (MatTek, Ashland, MA, USA) at a density of 4 × 10^4^ cells per dish. After 24 h, the medium was replaced with fresh medium. Cells were incubated with LysoTracker Red DND-99 (25 nM) for 30 min and then incubated with C6-labeled NC@PDA-NH_2_, NC, or free probe C6 for 2 h. Following washing with PBS, the cells were fixed with 4% paraformaldehyde in PBS for 20 min. After nucleus staining with DAPI, fluorescent images were taken with a Zeiss AXIO Observer Z1 confocal microscope (ZEISS LSM710, Zeiss, Athens, German). The fluorescence intensity of the cells was quantitatively analyzed using FCM analysis (BD FACSCalibur, Sparks, MD, USA) at the excitation wavelength (Ex) of 466 nm and emission wavelength (Em) of 504 nm using a Flow Cytometry System (BD FACSCalibur, Sparks, MD, USA).

For quantitative comparison of intracellular drug content, TC-1 cells were seeded in a 12-well plate at a density of 1.2 × 10^5^ cells per well. After overnight incubation, the cell culture medium was replaced with fresh medium containing NC@PDA-NH_2_, NC, or free imatinib (firstly dissolved in DMSO and then diluted in PBS) with the final concentration equivalent of imatinib at 120 μg/mL. After 4 h, cells were washed with PBS and collected for determination of the intracellular imatinib amount by HPLC as described in Section 2.3.

The cytotoxicity of NC@PDA-NH_2_, NC, or free IMN on TC-1 or SiHa cells over a range of imatinib concentrations for 72 h was measured using the standard MTT method. Half maximal inhibitory concentration (IC_50_) was calculated using GraphPad Prism 6 (La Jolla, CA, USA).

Apoptosis of TC-1 or SiHa cells induced by NC@PDA-NH_2_, NC, or free IMN after incubation for 48 h was compared using flow cytometry after standard Annexin V-FITC/propidium iodide (PI) staining.

### 2.6. Preparation and Characterization of NC@PDA-NH_2_/FG

NC@PDA-NH_2_/FG was prepared by dissolving an appropriate amount of Pluronic F127 in the corresponding nanocrystal suspension at 4 °C. NC-loaded F127 in situ hydrogel (NC/FG) was prepared similarly. Free imatinib/FG was prepared using the thin film-cold method as described in previous literature [10].

The rheology of NC@PDA-NH_2_/FG with three different F127 concentration was investigated using a rotatory rheometer (Bohlin Gemini Π, Malvern Instruments Ltd. Co., Malvern, UK) equipped with a parallel plate in the oscillation mode from 15–40 °C at a heating rate of 1 °C/min with a fixed frequency of 1 Hz and a fixed strain of 0.01. In vitro release of PC@PDA@NC/FG was evaluated using the membraneless method using vaginal fluid simulant (VFS) as the release medium. The concentration of imatinib in release samples was measured by HPLC as described in Section 2.3.

### 2.7. Ex Vivo and In Vivo Evaluation of the Mucoadhesiveness of NC@PDA-NH_2_/FG

For suitable gelation temperature, a weight concentration of 17.5% of F127 was selected for further investigation. Ex vivo adhesion on murine vaginal mucosa, intramucosal penetration of fluorescent-labeled NC@PDA-NH_2_/FG, NC/FG, and corresponding free probe/FG, and the intravaginal drug placement of NC@PDA-NH_2_/FG, NC/FG, and free IMN/FG were compared with methods similar to our previous work [10] in healthy C57BL/6 female mice.

For ex vivo adhesion evaluation, C57BL/6 female mice were sacrificed with excess inhalation of ether to get the vagina mucosa. Onto the freshly prepared vagina mucosa which was smoothed out and fixed on a plastic card, 10 μL of corresponding tested formulation was dropped with a pipette. These tissues were kept at 37 °C under repeated flushing with 10 µL of VFS every 10 min with a pipette. Near-infrared fluorescent (NIR) images were obtained every hour for 8 h with a whole-mouse fluorescent imaging system (IVIS spectrum, PerkinElmer, Santa Clara, CA, USA) at Ex 748 nm and Em 780 nm.

For in vivo intramucosal penetration, 10 µL of tested formulation per mouse was intravaginally administered to female ICR mice by a microliter syringe with a blunt needle. Three mice were randomly selected from each group and sacrificed with excess inhalation of ether to get the vaginal tissue which was fixed in paraformaldehyde for 24 h, dehydrated in sucrose solution, embedded in OCT, sectioned on a freezing microtome, stained with DAPI, and observed under a fluorescence microscope (DMI 4000, Leica Camera Co., Wetzlar, Germany).

For quantitation of intravaginal drug placement, healthy mice (24 mice per group) were intravaginally administered with NC@PDA-NH2/FG, NC/FG, or free drug (suspended imatinib powder)/FG, as described in Section 2.5. At every pre-determined time point (immediately, 2 h, 4 h, 6 h, and 8 h after administration), six mice were randomly selected from each group to perform vaginal lavage in which the vagina was thoroughly lavaged by normal saline as previously reported. Imatinib concentration in the vaginal lavage and mucosa was determined as described in Section 2.3.

### 2.8. In Vivo Efficacy against the Orthotopic Cervicovaginal Tumor Model in Mice

An orthotopic cervicovaginal TC-1 tumor model was established in female C57BL/6 mice according to the method of Yang et al. [26]. Mice (6–8 weeks old) were subcutaneously treated with medroxyprogesterone acetate (2.5 mg per mouse per day) for a consecutive seven days prior to tumor inoculation. Then, mice were anesthetized to gently disrupt their cervicovaginal epithelia with a cytobrush. TC-1 cells were inoculated by vaginal instillation (1 × 10^5^ cells per mouse) three days prior to day 0. Following day 0, intravaginal administration began. The formation and growth of tumor was preliminarily confirmed by palpation, as well as anatomical and histological examination, which found that TC-1 tumors grew along the length of the cervicovaginal tract and extended laterally toward surrounding tissues.

To compare the tumor growth inhibition effect of NC@PDA-NH_2_/FG vs. NC/FG at three imatinib dosages (2, 4, and 8 mg/kg), 160 tumor-bearing mice were divided into eight groups (20 mice per group) including NC@PDA-NH_2_/FG (8 mg/kg), NC@PDA-NH_2_/FG (4 mg/kg), NC@PDA-NH_2_/FG (2 mg/kg), NC/FG (8 mg/kg), NC/FG (4 mg/kg), NC/FG (2 mg/kg), free imatinib/FG (4 mg/kg), and normal saline intravaginally administered every other day. The animal number for each group was set at 20 to ensure that at least six animals survived by day 21 for all groups, especially for the normal saline control group. On day 21, mice were sacrificed for resection of uterus, cervix, and vagina to take photographs, weighing the tumors, and performing further microscopic examination for H&E staining or TUNEL assay (DeadEnd^TM^ Fluorometric TUNEL system, Promega, Madison, WI, USA). The change in the body weight was also monitored.

### 2.9. Statistical Analysis

All statistical analysis was performed with GraphPad Prism 6. Data were analyzed with one-way ANOVA test to determine the difference of means among groups. The logrank (Mantel-Cox) test was used to compare survival curves. A value of *p* < 0.05 was considered statistically significant.

## 3. Results and Discussion

### 3.1. Preparation and Characterization of NC@PDA-NH_2_

**Scheme 1:**
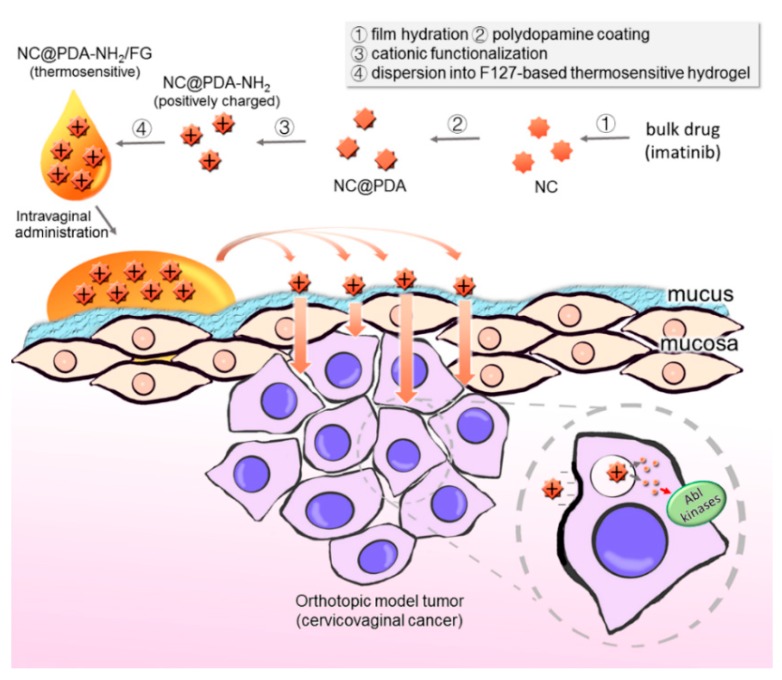
Illustration of the preparation and in vivo fate of amino-group-functionalized polydopamine (PDA)-coated imatinib nanocrystals (NC) dispersed in F127 (FG) hydrogel (NC@PDA-NH_2_/FG) in the orthotopic mouse cervicovaginal cancer model after intravaginal administration.

As depicted by Scheme 1, the core NCs were prepared by film hydration. Subsequently, dopamine was added to self-polymerize on the surface of NCs to obtain PDA-coated NCs (NC@PDA). The PDA coating provided the reacting platform for Boc-NH-CH_2_CH_2_-NH_2_. Finally, the Boc groups were removed by acidic treatment to provide positive charges on the surface of NC@PDA-NH_2_. NC@PDA-NH_2_ could be prepared with a relative high drug content of 76.84 ± 1.78% (Table 1).

**Figure 1:**
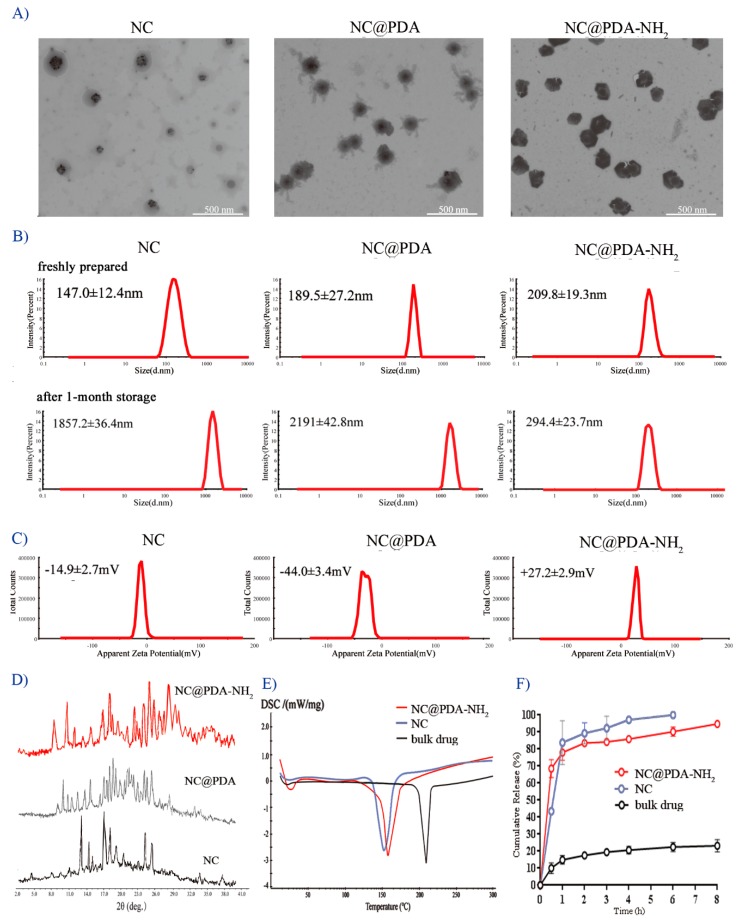
Characterization of NC, NC@PDA, and NC@PDA-NH_2_. (**A**) TEM images. (**B**) size distribution measured by dynamic light scattering (DLS). Upper panels: freshly prepared; lower panels: after one month of storage. (**C**) Zeta-potential measured by DLS. (**D**) X-ray diffraction (XRD) patterns. (**E**) Differential scanning calorimetry (DSC) curves. (**F**) In vitro release profiles in vaginal fluid simulant (VFS) (*n* = 3, mean ± SD).

NC, NC@PDA, and NC@PDA-NH_2_ all showed a near-spheroid shape (Figure 1A) with nanoscale size (Figure 1B). The zeta potentials of NC and NC@PDA were −14.9 ± 2.7 mV and −44.0 ± 3.4 mV, respectively, while that of NC@PDA-NH_2_ was +27.2 ± 2.9 mV (Figure 1C), indicating the success of cationic modification. The size distribution of NC@PDA-NH_2_ remained basically unchanged after one month, while the size of unmodified NC increased after only several days, which may be attributed to the electrostatic expulsion among positively charged NC@PDA-NH_2_.

The XRPD patterns of bulk drug displayed peaks at 12.96°, 14.13°, 17.1°, 18.7°, 20.9°, 24.4°, and 25.2° (2θ), consistent with previous reports for I-type crystalline imatinib. By contrast, the patterns of NC and NC@PDA-NH_2_ both showed distinct peaks at 15.0°, 17.2°, 18.1°, 18.7°, and 21.1°, characteristic of G-type crystalline [27] (Figure 1D). The DSC curves also indicated changes in the crystalline type from bulk drug to nanocrystals (Figure 1E).

In vitro release from NC and NC@PDA-NH_2_ was much faster than that of bulk drug, perhaps related to their small size. This may be beneficial because most of the imatinib could remain in nanocrystals during the penetration across the mucus, as well as upon interaction with mucosa and entering tumor cells.

### 3.2. Cationic Functionalization Favored Interaction of Nanocrystals with Mucin

**Figure 2:**
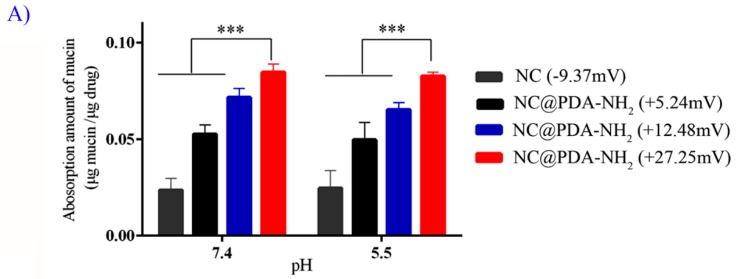

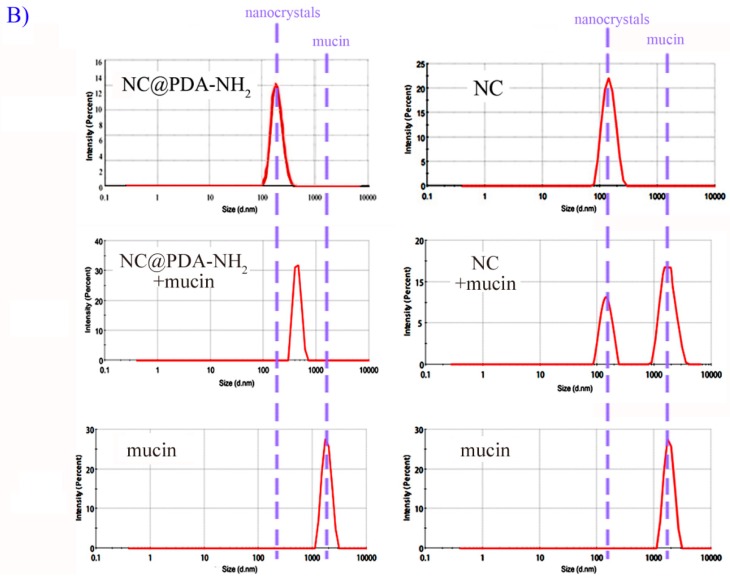
Interaction between NC@PDA-NH_2_ and mucin. (**A**) Adsorption of mucin by NC@PDA-NH_2_ with different surface charges or NC (*n* = 3, mean ± SD). (**B**) Change in size distribution of NC@PDA-NH_2_ (or NC) after mixing with mucin. *** indicates *p* < 0.001.

Adsorption of mucin is a widely used parameter in the evaluation of mucoadhesiveness [28]. NC@PDA-NH_2_ with different positive surface charges was prepared (Figure S2, Supplementary Materials) and compared with respect to the capability of adsorbing mucin (Figure 2A). It is clear that the amount of mucin adsorption was directly correlated with the value of positive surface charge, indicating the important role of cationic functionalization in mucin adsorption by NC@PDA-NH_2_. The interaction between NC@PDA-NH_2_ and mucin was further proven by the increase in size after mixing NC@PDA-NH_2_ and mucin (Figure 2B). A similar change in the particle size of mucin was also observed in the presence of positively charged polymers, including chitosan and amino-functionalized poloxamer 407, indicative of the interaction between positively charged polymers and mucin [29].

### 3.3. Cationic Functionalization Favored Interaction of Nanocrystals with Cells

**Figure 3:**
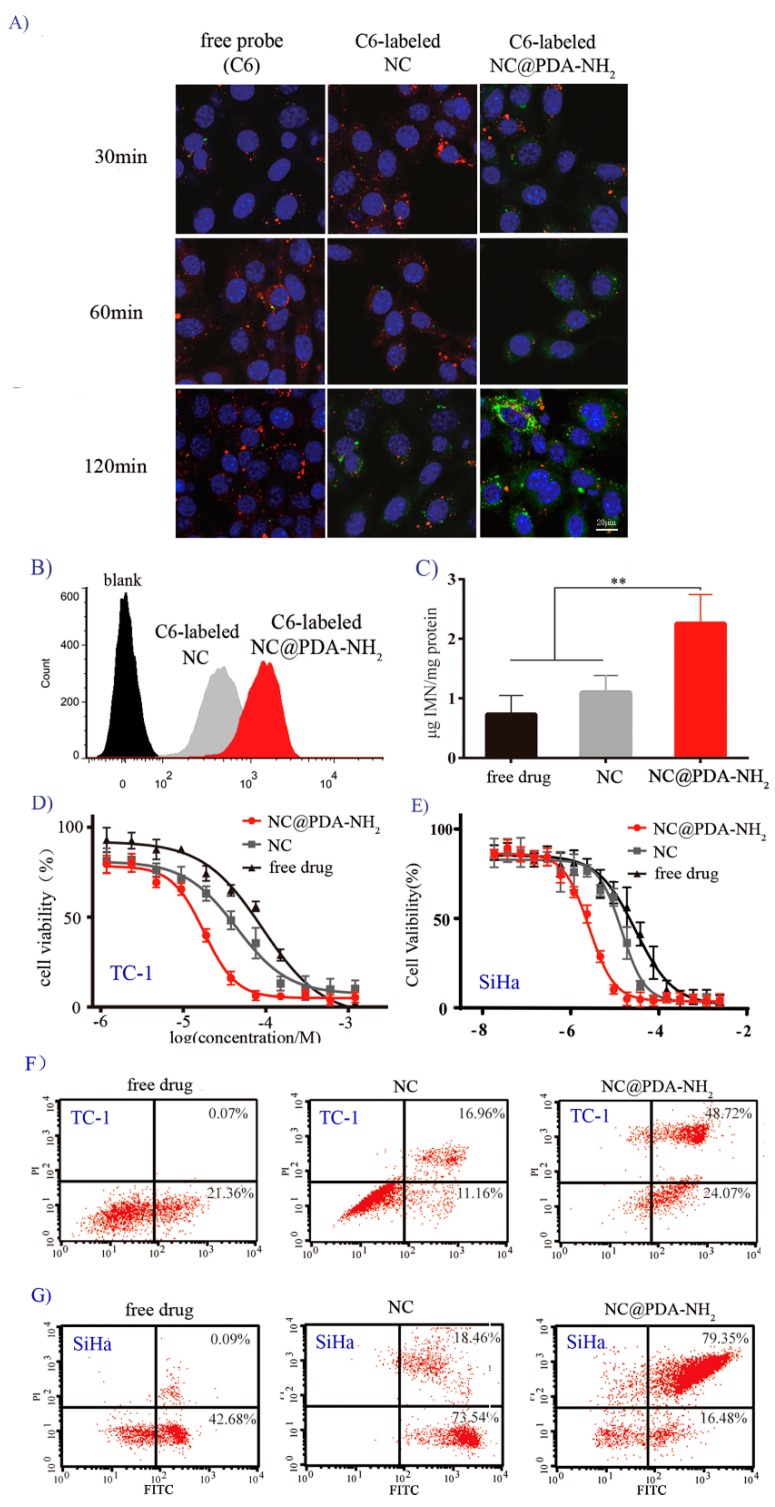
Cellular uptake and cytotoxicity in cervical cancer-related cell lines. (**A**) Representative CLSM images of TC-1 cells co-incubated with 6-coumarin (C6)-labeled NC@PDA-NH_2_, NC, or free probe C6 (green signal). The nucleus was stained with 2-(4-amidinophenyl)-6-indolecarbamindine dihydrochloride (DAPI; blue) and the lysosomes were stained with LysoTracker Red. (**B**) Flow cytometry analysis on the fluorescent intensity of cells after co-incubation for 2 h with C6-labeled NC@PDA-NH_2_, NC, or free probe in TC-1 cells. (**C**) Intracellular drug content after co-incubation for 72 h with NC@PDA-NH_2_, NC, or free imatinib in TC-1 cells (*n* = 5, mean ± SD, ** represents *p* < 0.01). (**D**,**E**) In vitro cytotoxicity of NC@PDA-NH_2_, NC, or free imatinib after 48 h of co-incubation in TC-1 cells (**D**) or SiHa cells (**E**). (**F**,**G**) Apoptotic cell populations determined by flow cytometry with Annexin V-FITC and propidium iodide (PI) staining after co-incubation with NC@PDA-NH_2_, NC, or free imatinib in TC-1 cells (**E**) or SiHa cells (**G**). The lower-left (Q3), lower-right (Q4), upper-right (Q2), and upper-left (Q1) quadrants in each panel indicate the populations of normal, early, and late apoptotic, and apoptotic necrotic cells, respectively.

Investigation into the interaction between NC@PDA-NH_2_ and cervical cancer-related cells was performed on TC-1 cells, which are immortalized murine epithelial cells transformed to express human papillomavirus (HPV)-16 E6/E7 and activated human c-Ha-ras oncogene, and exhibit similar genetic traits to human papillomavirus (HPV)-induced cervical tumors [26]. Both CLSM images (Figure 3A) and flow cytometry data (Figure 3B) demonstrated more cellular uptake of NC@PDA-NH_2_ than NC, attributable to the electrostatic interaction between the positive charges on NC@PDA-NH_2_ and the negative charges on the cell membrane. The intracellular fluorescent signal of NC@PDA-NH_2_ overlapped with that of LysoTracker with a Pearson’s correlation coefficient (*R*) of 0.80, suggesting possible involvement of the endocytosis pathway. Further investigation into the cellular uptake mechanism will be included in future work. Quantitative measurements of intracellular IMN concentration further confirmed that more imatinib entered cells after incubation with NC@PDA-NH_2_ than NC or free imatinib (Figure 3C). The above enhanced cellular uptake was in accordance with the prolonged adhesion on the surface of cellular monolayers of positively charged liposomes than negatively charged or neutral liposomes [30].

As shown by the MTT results (Figure 3D), enhanced cellular growth inhibition was observed with NC@PDA-NH_2_. The IC_50_ values of NC@PDA-NH_2_, NC, and free drug were 16.8 μM, 39.3 μM, and 84.9 μM, respectively. Such enhanced cellular growth inhibition of NC@PDA-NH2 compared to NC and free drug might be relevant with the different uptake and, thus, different intracellular drug accumulation.

More cellular apoptosis was also observed with NC@PDA-NH_2_. NC@PDA-NH_2_, NC, and free drug resulted in 24.07%, 11.16%, and 21.36% early apoptotic cells in the fourth quadrant (Q4) and 48.7%, 16.96%, and 0.07% late apoptotic cells in Q1, respectively. Similar trends were also observed in MTT and apoptosis results with another cervical cancer-related cell line (SiHa cells).

### 3.4. Cationic Functionalization Prolonged Intravaginal Retention and Enhanced Mucosal Penetration

Before ex vivo and in vivo application, NC@PDA-NH_2_ was dispersed in Pluronic F127-based thermosensitive hydrogel (FG) to avoid leakage which might happen if administered as a simple aqueous dispersion, and enough time was allowed for nanocrystals to interact with the mucosa. Other vehicles for nanoparticles were also reported for vaginal use, such as hydroxypropyl methylcellulose/poly(vinyl alcohol) films [31] and poly(vinyl alcohol) nanofibers [32]. FG was also applied as a vaginal formulation vehicle with good safety for many therapeutic agents. Due to its lack of mucoadhesive properties, FG may serve as an ideal vehicle for the evaluation of NC@PDA-NH_2_.

**Figure 4:**
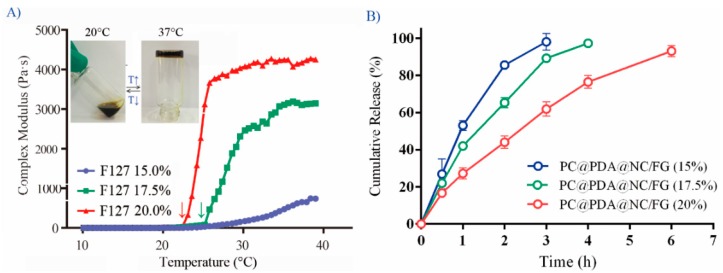
Rheological and in vitro release profiles of NC@PDA-NH_2_/FG. (**A**) Temperature-dependent rheological profiles of NC@PDA-NH_2_/FG with different F127 concentration (15.0%, 17.5%, and 20.0%, *w*/*w*). The red and green arrows denote the gelation temperature for 20.0% and 17.5%, respectively, while the thermal phase transition was not obvious for 15.0%. The insert is the typical photograph of NC@PDA-NH_2_/FG (17.5%) at storage (20 °C) or body temperature (37 °C). (**B**) Release profiles from NC@PDA-NH_2_/FG at different F127 concentration (15.0%, 17.5% and 20.0%, *w*/*w*) at 37 °C in VFS (*n* = 3, mean ± SD).

Consistent with previous reports on FG [33], the phase transition temperature and in vitro drug release rate of NC@PDA-NH_2_-containing FG (NC@PDA-NH_2_/FG) were both dependent on the concentration of Pluronic F127 (Figure 4). A concentration of 17.5% (*w*/*w*) was selected for Pluronic F127 to assure easy administration and moderate dispersion into vaginal fluid.

**Figure 5:**
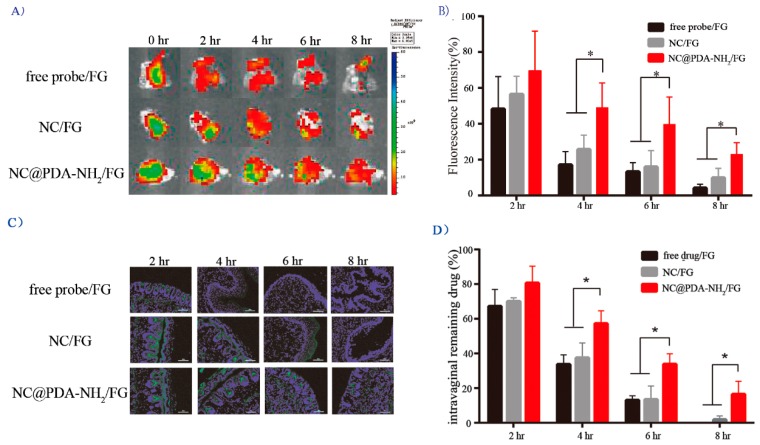
Ex vivo mucoadhesiveness and in vivo placement. (**A**) Typical fluorescent images, and (**B**) semi-quantitative fluorescent intensity (*n* = 6, mean ± SD) for the ex vivo residence of 1,1′-dioctadecyl-3,3,3′,3′-tetramethylindotricarbocyanine iodide (DiR)-labeled NC@PDA-NH_2_/FG, NC/FG, or free probe DiR/FG on the surface of ex vivo vaginal mucosa of healthy mice during flushing by VFS at 37 °C. (**C**) Fluorescent microscopic images (green signal for C6, blue signal for DAPI; scale bar = 100 μm), and (**D**) intravaginal residence percentage of imatinib in healthy mice vaginally administered with NC@PDA-NH_2_/FG, NC/FG, or free imatinib/FG (*n* = 6, mean ± SD); * indicates *p* < 0.05.

When dropped on ex vivo murine vagina mucosa and continuously flushed with VFS for 8 h, NC@PDA-NH_2_/FG resulted in significantly longer residence of fluorescent signal than NC/FG or free probe/FG (Figure 5A,B). When observed microscopically, prolonged, more, and deeper fluorescence was found in the vaginal mucosa of mice administered with NC@PDA-NH_2_/FG, suggesting improved accessibility and penetration into vaginal mucosa. Quantitative measurements of intravaginal imatinib amount further confirmed the superiority of NC@PDA-NH_2_/FG with respect to intravaginal residence (Figure 5D).

### 3.5. Cationic Functionalization Improved Tumor Inhibition in the Orthotopic TC-1 Cervical Cancer Model

The orthotopic TC-1 model was reported to represent a stringent model for cervicovaginal cancer within a short time frame and localized similarly to the anatomy of cervical tumors. In addition, this model is suitable for testing mucoadhesive formulations because it features the cervicovaginal mucus layer that acts as a barrier to particle penetration [26].

**Figure 6:**
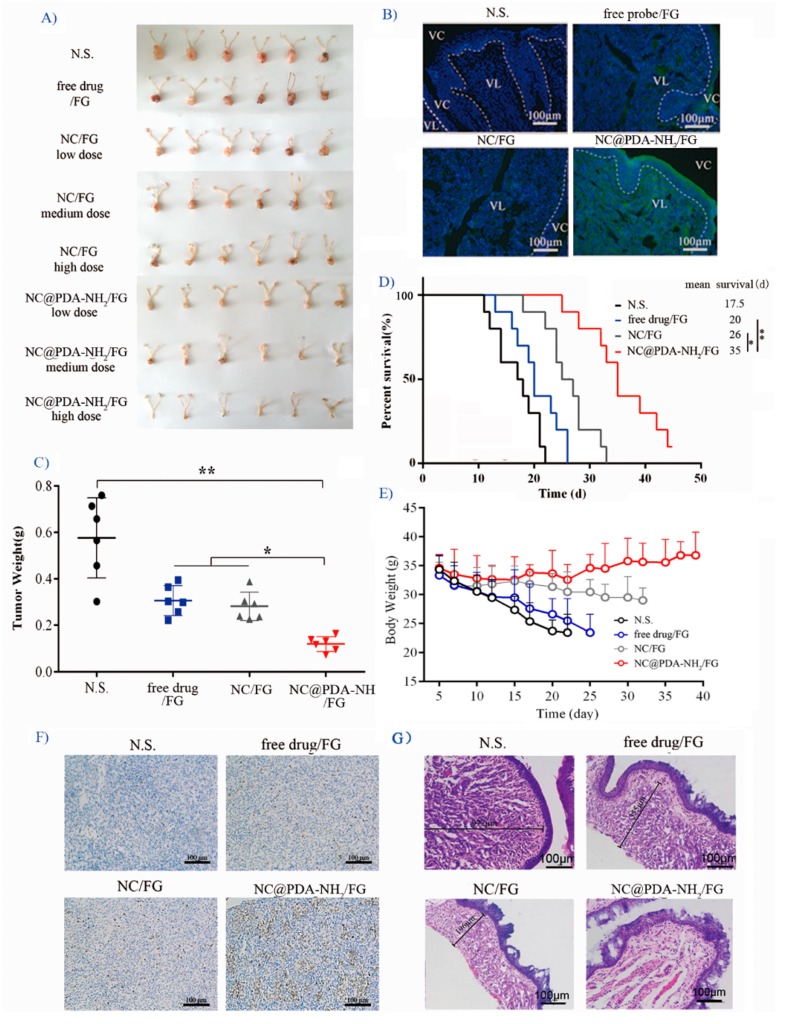
In vivo evaluation in orthotopic TC-1 cervicovaginal model tumor-bearing mice after intravaginal administration of different formulations every other day. (**A**) Dose-dependent inhibition on the tumor size after three-week treatment (dose: 2, 4, and 8 mg/kg/2 d). (**B**) Penetration of fluorescent signal into the vaginal mucosa (4 h after administration). (**C**) Tumor weights after treatment for three weeks (dose: 4 mg/kg/2 d; *n* = 6). (**D**) Survival time (dose: 4 mg/kg/2 d; *n* = 10). (**E**) Change in anima body weight (dose: 4 mg/kg/2 d; *n* = 10). (**F**) Representative immunohistochemical microphotographs of tumor sections stained for apoptosis after three-week treatment (dose: 4 mg/kg/2 d). (**G**) Representative H&E microphotographs of tumors and vagina after three-week treatment (dose: 4 mg/kg/2 d). * indicates *p* < 0.05, ** indicates *p* < 0.01.

The tumor growth inhibition effect was firstly investigated after daily intravaginal administration treatment with NC@PDA-NH_2_/FG or NC/FG for three weeks at three different doses (2, 4, and 8 mg/kg/2 d) by observing the tumor size by the end (Figure 6A). The inhibition effects of NC@PDA-NH_2_/FG and NC/FG were both dose-dependent. More importantly, NC@PDA-NH_2_/FG inhibited the growth of model tumors more effectively. This might be explained by more accessibility of NC@PDA-NH_2_ to the vaginal mucosal as indicated by the semi-quantitative analysis of optical density of fluorescent microscopic images.

More and deeper fluorescence was found in the vaginal mucosa bearing model tumors for fluorescent-labeled NC@PDA-NH_2_/FG (Figure 6B). Based on the Kaplan–Meier survival curves at the dosage of 4 mg/kg/2 d, NC@PDA-NH_2_/FG prolonged the median survival time, 7.0-fold that of free drug/FG (35 d vs. 20 d) and 2.1-fold that of NC/FG group (35 d vs. 26 d). More apoptosis areas were also observed in the microscopic sections from the NC@PDA-NH_2_/FG group than the NC/FG group (Figure 6E).

The safety of NC@PDA-NH_2_/FG was evaluated by monitoring the change in the animal body weight during the abovementioned three-week treatment and histological examination of the vaginal mucosa by the end. As shown in Figure 6E, the body weight of mice in the NC@PDA-NH_2_/FG group slowly increased, reflecting their relatively good health state. By contrast, the body weight continuously decreased in the free drug/FG or control groups (normal saline).

H&E staining sections (Figure 6F) also demonstrated the maximal anti-tumor efficiency and minimal mucosal toxicity for the NC@PDA-NH_2_/FG group. The mucosal epithelium was destructed and compressed by the prosperous tumor tissues in the control group, while the mucosal epithelium partially recovered in the NC@PDA-NH_2_/FG group.

As the TC-1 model tumor grew, elevated interstitial pressure within tumor tissue impaired intratumor penetration and made complete tumor eradication ultimately difficult for free drug [34]. It is encouraging that NC@PDA-NH_2_/FG effectively suppressed the growth of TC-1 model tumors. Future studies using other murine cervical tumor models which are less aggressive and resemble the slow tumor progression in humans will further substantiate the effectiveness of NC@PDA-NH_2_/FG.

## 4. Conclusions

In summary, positively charged NC@PDA-NH_2_ significantly interacted with mucin in vitro and resulted in more cellular uptake, cellular growth inhibition efficacy, and induction of apoptosis. NC@PDA-NH_2_/FG exhibited good ex vivo and in vivo mucoadhesiveness, as well as improved anti-tumor efficacy in the murine orthotopic cervicovaginal cancer tumor model. In conclusion, cationic functionalization endowed imatinib nanocrystals good potential as a novel mucoadhesive approach for intravaginal treatment of cervicovaginal cancer. Our work also reveals the good potential of surface functionalization based on polydopamine coating on nanocrystals, which appear to be a good nanoscale drug delivery system with high drug content for poorly soluble drugs.

## Figures and Tables

**Table 1:** Drug loading (DL) and entrapment efficiency (EE) for nanocrystal (NC), polydopamine (PDA)-coated NC (NC@PDA), and amino-group-functionalized NC@PDA (NC@PDA-NH_2_) (*n* = 3, mean ± SD).

	Drug Loading (DL) (%)	Entrapment Efficiency (EE) (%)
NC	94.47 ± 2.85	94.45 ± 4.81
NC@PDA	82.72 ± 3.66	83.55 ± 12.01
NC@PDA-NH2	76.84 ± 1.78	87.11 ± 11.43

## Supplementary Materials

The following are available online at http://www.mdpi.com/1999-4923/11/1/15/s1, Figure S1: Influence of reaction time on the size distribution and zeta potential of resulted nanocrystals measured by dynamic light scattering. Left: reaction time of PDA@NC with Boc-NHCH_2_CH_2_NH_2_; right: deprotection time in acidic environment, Figure S2: NC@PDA-NH2 with different zeta potentials for comparison of mucin adsorption, Figure S3: Size distribution and zeta potentials of C6-labeled NC, NC@PDA, and NC@PDA-NH2, Figure S4: Typical chromatograph for imatinib (1 ug/mL, LLOQ), Figure S5: Typical chromatograph of vaginal lavage samples after intravaginal administration of NC@PDA-NH2/FG.

## Abbreviations

PDA	polydopamine
NC	nanocrystals
NC@PDA-NH2	cationic functionalized polydopamine-coated imatinib nanocrystals
NC@PDA	polydopamine-coated imatinib nanocrystals
F127	Pluronic F127
FG	Pluronic F127-based in situ hydrogel
TKI	tyrosine kinase inhibitors
IMN	imatinib
C6	6-coumarin
DiR	1,1′-dioctadecyl-3,3,3′,3′-tetramethylindotricarbocyanine iodide
OCT	optical coherence tomography
DAPI	2-(4-amidinophenyl)-6-indolecarbamindine dihydrochloride
PGM	porcine gastric mucin
DL	drug loading
EE	encapsulation efficiency
TEM	transmission electron microscope
DLS	dynamic light scattering
XRPD	X-ray powder diffraction
DSC	differential scanning calorimetry
VFS	vaginal fluid stimulant
