# Correction: Ci et al. Enhanced Delivery of Imatinib into Vaginal Mucosa via a New Positively Charged Nanocrystal-Loaded in Situ Hydrogel Formulation for Treatment of Cervical Cancer. *Pharmaceutics* 2019, *11*, 15

**DOI:** 10.3390/pharmaceutics15092188

**Published:** 2023-08-24

**Authors:** Li-qian Ci, Zhi-gang Huang, Feng-mei Lv, Jun Wang, Ling-lin Feng, Feng Sun, Shui-juan Cao, Zhe-peng Liu, Yu Liu, Gang Wei, Wei-yue Lu

**Affiliations:** 1 Department of Pharmaceutics, School of Pharmacy, Fudan University & Key Laboratory of Smart Drug Delivery (Fudan University), Ministry of Education, Shanghai 201203, China; 2 School of Medical Instrument and Food Engineering, University of Shanghai for Science and Technology, Shanghai 200093, China; 3 NHC Key Laboratory of Reproduction Regulation (Shanghai Institute of Planned Parenthood Research), Fudan University, Room 904, No 1 Research Building, 2140 Xietu Road, Shanghai 200032, China; 4 Chinese Academy of Sciences Shanghai Institute of Materia Medica, Shanghai 201203, China; 5 Experimental Teaching Center, School of Pharmacy, Fudan University, Shanghai 201203, China

## 1. Error in Figure

In the original publication [1], there was a mistake in Figure 3A as published. When conducting fluorescent photographing, more than one pictures were taken at the beginning for adjust the fluorescent setting parameters using the cells incubated with free probes. During the preparation of the figure, all the figures were put in one file for convenience of batch processing and a photograph from cells incubated with free probes was mistakenly used as the NC-120 min. The corrected Figure 3 appears below. 

In the original publication [1], there was also a mistake in Figure 6G as published. When preparing microscopy sections, more than one section was obtained from every animal. A mislabel of another section from the same mice treated with free drug/FG as NC/FG. The corrected Figure 6G appears below. 

The authors state that the scientific conclusions are unaffected. This correction was approved by the Academic Editor. The original publication has also been updated.

## Figures and Tables

**Figure 3:**
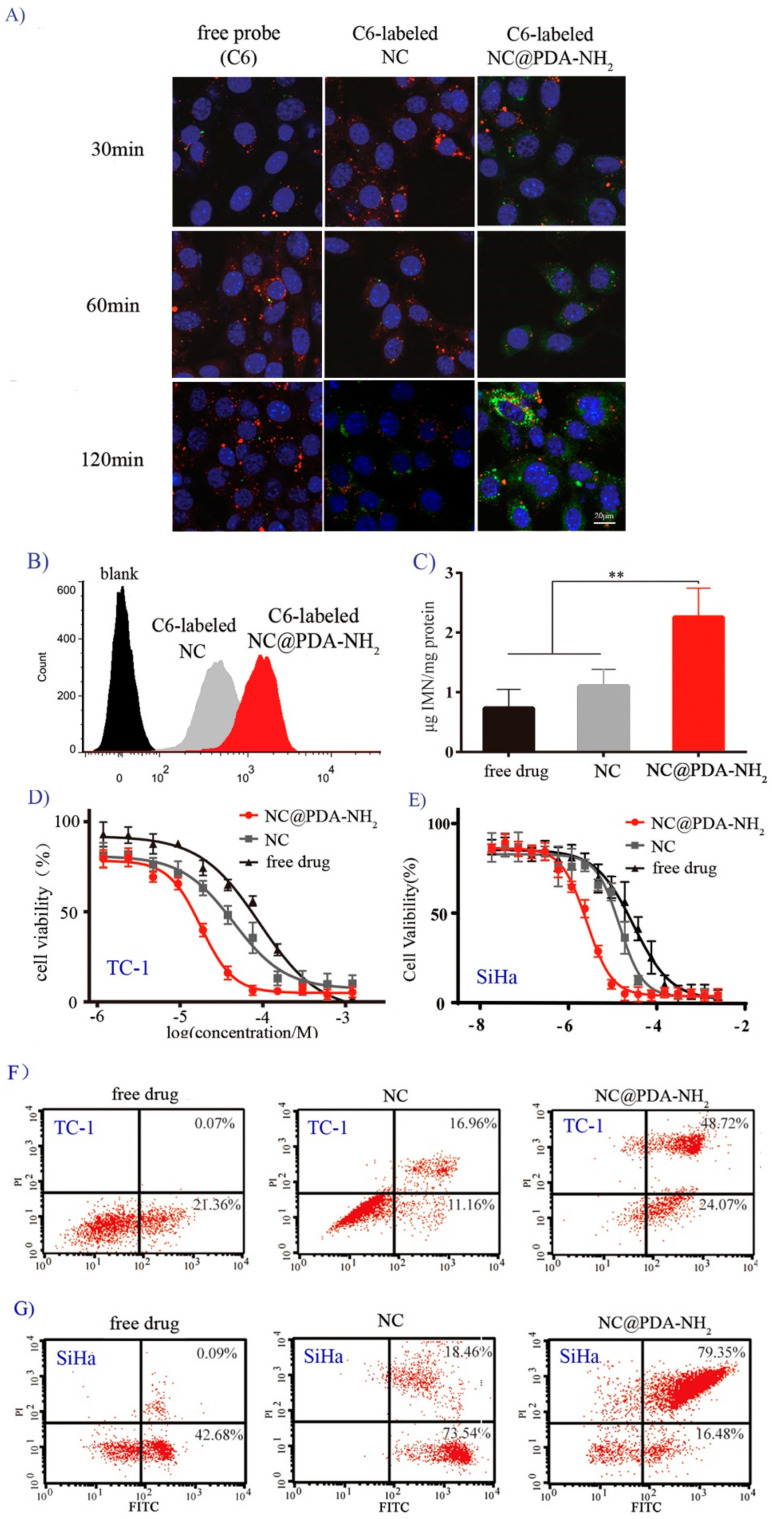
Cellular uptake and cytotoxicity in cervical cancer-related cell lines. (**A**) Representative CLSM images of TC-1 cells co-incubated with 6-coumarin (C6)-labeled NC@PDA-NH_2_, NC, or free probe C6 (green signal). The nucleus was stained with 2-(4-amidinophenyl)-6-indolecarbamindine dihydrochloride (DAPI; blue) and the lysosomes were stained with LysoTracker Red. (**B**) Flow cytometry analysis on the fluorescent intensity of cells after co-incubation for 2 h with C6-labeled NC@PDA-NH_2_, NC, or free probe in TC-1 cells. (**C**) Intracellular drug content after co-incubation for 72 h with NC@PDA-NH_2_, NC, or free imatinib in TC-1 cells (*n* = 5, mean ± SD, ** represents *p* < 0.01). (**D**,**E**) In vitro cytotoxicity of NC@PDA-NH_2_, NC, or free imatinib after 48 h of co-incubation in TC-1 cells (**D**) or SiHa cells (**E**). (**F**,**G**) Apoptotic cell populations determined by flow cytometry with Annexin V-FITC and propidium iodide (PI) staining after co-incubation with NC@PDA-NH_2_, NC, or free imatinib in TC-1 cells (**E**) or SiHa cells (**G**). The lower-left (Q3), lower-right (Q4), upper-right (Q2), and upper-left (Q1) quadrants in each panel indicate the populations of normal, early, and late apoptotic, and apoptotic necrotic cells, respectively.

**Figure 6:**
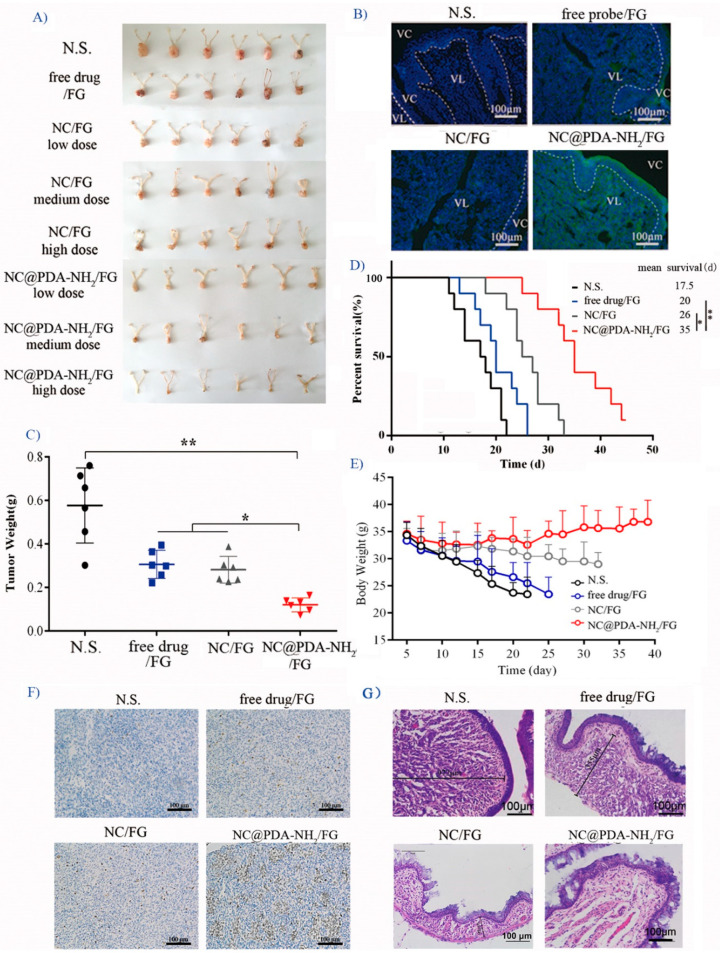
In vivo evaluation in orthotopic TC-1 cervicovaginal model tumor-bearing mice after intravaginal administration of different formulations every other day. (**A**) Dose-dependent inhibition on the tumor size after three-week treatment (dose: 2, 4, and 8 mg/kg/2 d). (**B**) Penetration of fluorescent signal into the vaginal mucosa (4 h after administration). (**C**) Tumor weights after treatment for three weeks (dose: 4 mg/kg/2 d; *n* = 6). (**D**) Survival time (dose: 4 mg/kg/2 d; *n* = 10). (**E**) Change in anima body weight (dose: 4 mg/kg/2 d; *n* = 10). (**F**) Representative immunohistochemical microphotographs of tumor sections stained for apoptosis after three-week treatment (dose: 4 mg/kg/2 d). (**G**) Representative H&E microphotographs of tumors and vagina after three-week treatment (dose: 4 mg/kg/2 d). * indicates *p* < 0.05, ** indicates *p* < 0.01.
