# Supplementary material for: Enhanced Delivery of Imatinib into Vaginal Mucosa via a New Positively Charged Nanocrystal-Loaded in Situ Hydrogel Formulation for Treatment of Cervical Cancer
Source: Pharmaceutics. 2019 Jan 4;11(1):15. doi: 10.3390/pharmaceutics11010015 (PMC6359353; doi:10.3390/pharmaceutics11010015)
Supplement: Supplementary file 1 [file pharmaceutics-11-00015-s001.pdf]

pharmaceutics

MDPI

Supplementary Materials: Enhanced Delivery of Imatinib into Vaginal Mucosa via a New Positively Charged Nanocrystal-Loaded in Situ Hydrogel Formulation for Treatment of Cervical Cancer

Li-qian Ci, Zhi-gang Huang, Feng-mei Lv, Jun Wang, Ling-lin Feng, Feng Sun, Shui-juan Cao, Zhe-peng Liu, Yu Liu, Gang Wei and Wei-yue Lu

1. Optimization of the reaction conditions in the third step

Different reaction times for NC@PDA with Boc-$NHCH_2CH_2NH_2$ and different deprotection times in the acidic environment were tested to optimization for adequate positive charges. Figure S1 shows that the reaction time of NC@PDA with Boc-$NHCH_2CH_2NH_2$ should be not less than three hours and the deprotection time should be not less than three hours.

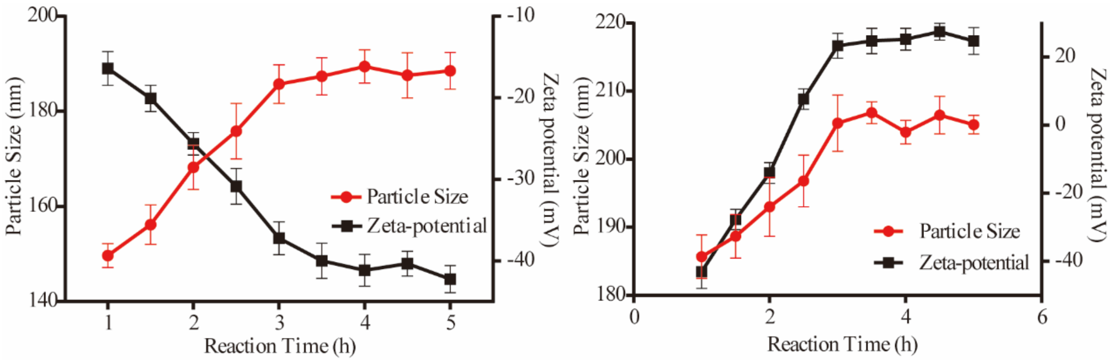

Figure S1. Influence of reaction time on the size distribution and zeta potential of resulted nanocrystals measured by dynamic light scattering. Left: reaction time of PDA@NC with Boc-$NHCH_2CH_2NH_2$; right: deprotection time in acidic environment.

2. Preparation of NC@PDA-NH_2 with different zeta-potential values

To investigate the influence of positive potential value on mucin adsorption, NC@PDA-NH_2 with different zeta-potential values was prepared by adding appropriate amounts of Boc-$NHCH_2CH_2NH_2$ in step 3. The zeta potentials of different NC@PDA-NH_2 are shown in Figure S2.

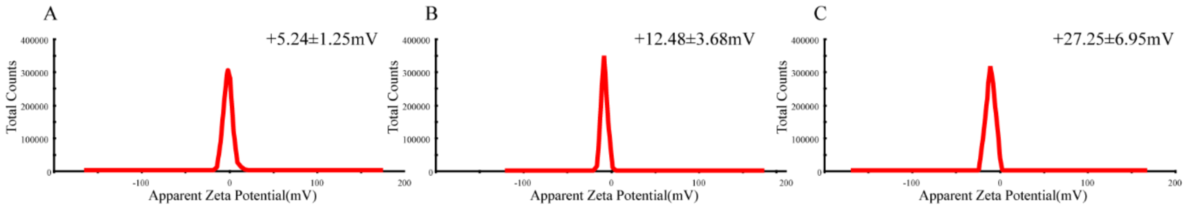

Figure S2. NC@PDA-NH_2 with different zeta potentials for comparison of mucin adsorption.

3. Size distribution and zeta potential of C6-labeled NC@PDA-NH_2

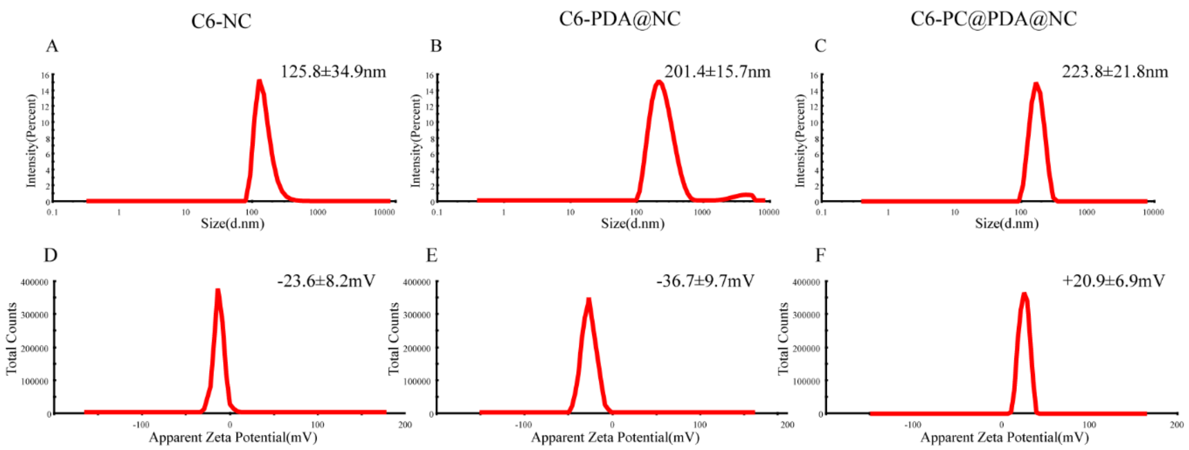

Figure S3. Size distribution and zeta potentials of C6-labeled NC, NC@PDA, and NC@PDA-NH_2.

4. HPLC calibration curves and linear range.

The linear range was 1–500 ug/mL. Typical chromatographs are shown in Figures S4 and S5. The retention time of IMN was ~7.3 min.

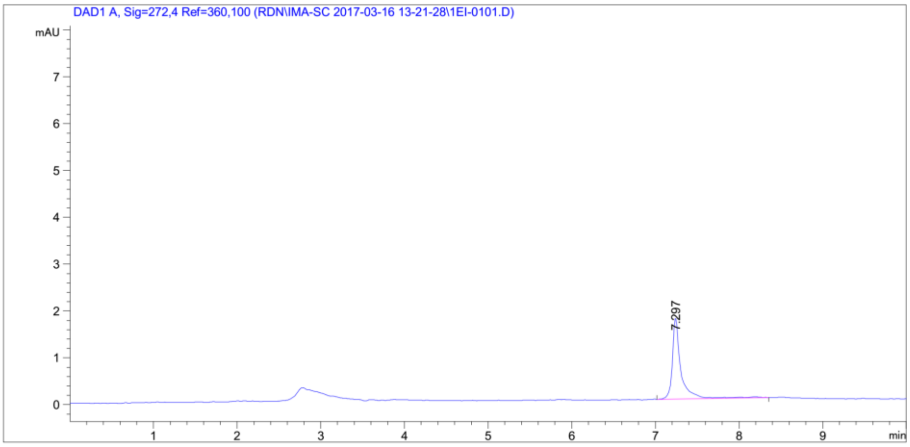

Figure S4. Typical chromatograph for imatinib (1 ug/mL, LLOQ).

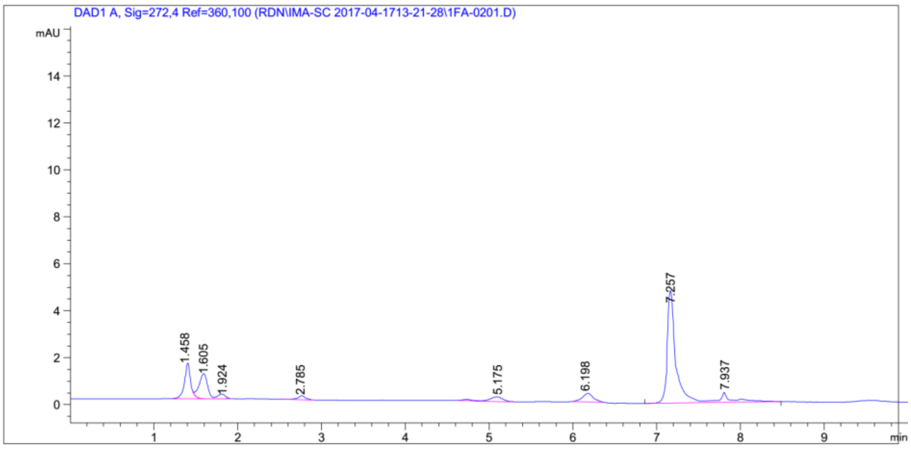

Figure S5. Typical chromatograph of vaginal lavage samples after intravaginal administration of NC@PDA-NH_2/FG.
